# CCR5 Inhibitors and HIV-1 Infection

**DOI:** 10.33696/AIDS.1.001

**Published:** 2019

**Authors:** Olga S. Latinovic, Marvin Reitz, Alonso Heredia

**Affiliations:** 1 Institute of Human Virology, University of Maryland School of Medicine, Baltimore, Maryland, USA; 2 Department of Microbiology and Immunology, University of Maryland School of Medicine, Baltimore, Maryland, USA; 3 School of Medicine, University of Maryland School of Medicine, Baltimore, Maryland, USA

## Introduction

Cellular components are attractive targets for antiretroviral therapy because they do not mutate as readily as do viral proteins do [1–3]. The identification of CCR5 as an HIV-1 coreceptor [4–7], facilitated by the discovery of the antiretroviral activities of CCR5 ligand β-chemokines [8], resulted in the development of new viral entry inhibitors to block CCR5 binding, including both- small molecules and CCR5 antibodies. In clinical trials of HIV-1 patients infected with CCR5-tropic HIV-1 only (R5 strains), these agents have achieved remarkable viral suppression by inhibiting HIV-1 entry and subsequent infection [9,10–14].

### CCR5 Coreceptor as an Antiretroviral Target and the Delta 32 Mutation

The CCR5 viral coreceptor, one of a family of chemokine receptors belonging to the G protein-coupled receptor family [15], is expressed on a variety of cell types, including activated T lymphocytes, macrophages and dendritic cells [16]. These receptors consist of seven transmembrane helices, an extracellular N-terminus, three extracellular loops (ECLs) and intracellular C-terminus. Elements located in the N-terminus and second ECL of CCR5 are specifically relevant for interactions with HIV-1 during virus entry, putting focus on them as attractive targets for designing more productive antiretroviral therapies. In addition, CCR5 has a further advantage as a cellular target because it is relatively unnecessary for normal immune function, in contrast with receptor CD4 and the viral coreceptor CXCR4 [17]. Both have critical roles in immune function [18,19], which severely limits their utility as antiretroviral therapy targets. The relative dispensability of CCR5 coreceptor is demonstrated in individuals homozygous for the Δ32 mutation of CCR5. These people are highly resistant to HIV-1 infection [20,21]. In addition, Δ32 heterozygous individuals’ progress to AIDS more slowly than do homozygous with the wild-type gene [22,23]. Moreover, CCR5 density levels (molecules/cell) on CD4^**+**^ T cells positively correlate with RNA viral loads [24] and progression to AIDS [25] in untreated infected individuals. The direct impact of CCR5 surface density on the antiretroviral activity of CCR5 antagonists has also been clearly established *in vitro* where CCR5 levels inversely correlate with rates of HIV-1 entry inhibition [26,27], especially by entry inhibitors [16, 22]. These findings, along with the apparent curative effect seen when Δ32 homozygous hematopoietic stem cells were transplanted into a patient with AIDS and leukemia (the “Berlin patient” study) [28], have given great stimulus for the use of CCR5 blockers for inhibiting HIV-1 entry and infection. It has led to extensive efforts to develop effective antiretroviral CCR5 inhibitors. These now include CCR5 antagonists [12, 29, 30, 31], fusion proteins that target the CCR5 N-terminus and other relevant sites in CCR5 [32], CCR5 antibodies [33, 34], and even drugs to reduce the surface density of CCR5 numbers. Some of these CCR5 blockers have achieved remarkable suppression of HIV-1 entry in clinical trials and clinical settings *in vivo* [12,29,34,35]. Entry inhibitors overall have a further appeal as antiretroviral agents, in that they immobilize HIV-1 within the extracellular environment, where it is accessible to the immune system [36].

### CCR5 Inhibitors

Several small-molecule CCR5 inhibitors have been developed in the last decade [37,38]. At present, the small-molecule CCR5 antagonist Maraviroc (MVC) is the only licensed CCR5 inhibitor on the market (Pfizer, 2007) [39] and is approved for use in treatment-naïve and treatment-experienced patients. It acts as an allosteric, non-competitive inhibitor of the receptor [40,41]. MVC is licensed for patients infected with only CCR5-tropic HIV-1 [42]. Oral administration of MVC has resulted in dramatic reductions in viral loads [42,43]. MVC and other small molecules have great *in vitro* synergy with other CCR5 blockers, including CCR5 monoclonal antibodies (mAbs) [33–35,43,44], significantly inhibiting HIV-1 entry into physiologically relevant primary cells *in vitro*.

Two other CCR5 inhibitors reached clinical trial phases, but both were discontinued for the different reasons. Aplaviroc (APL) administration gave significant reduction of plasma HIV-1 RNA copies during the first ten days of treatment [45], but development was terminated after reversible drug-induced hepatitis occurred in five subjects in phase II and III trials [46]. The other CCR5 antagonist, Vicriviroc (VCV), showed significant suppression of HIV-1 in combination with an optimized background regimen in placebo-controlled phase II studies in HIV treated patients, but increased rates of virologic failure in treatment-naive patients compared with an Efavirenz control arm led to the termination of a preceding phase II study [47–50].

Cenicriviroc (CVC), an experimental drug candidate for blocking CCR5 receptors, is in the phase III clinical trials [51]. Like MVC, this drug is a small-molecule CCR5 antagonist, but with a longer biological half-life than MVC. Both CCR5 inhibitors show beneficial pharmacokinetics and substantial reductions of plasma HIV-1 RNA load in HIV infected patients. It was suggested that the dosage of CVC (50–75 mg, QD, orally) may need adjustment. CVC also has additional activity as a CCR2 antagonist.

Resistance to MVC has been reported previously [52–54], and is due to three separate mechanisms. One mechanism involves selection of pre-existent minor HIV-1 variants that use CXCR4 as a coreceptor to enter target cells [55]. The second mechanism involves selection for mutants that can use inhibitor-bound CCR5 for entry [56]. The third mechanism involves selection for mutations, primarily in the V3 loop of gp120, which changes coreceptor use from CCR5 to CXCR4. The latter has been demonstrated *in vitro* [57], but is rare in infected patients treated with MVC [42].

Lastly, other alternative ongoing efforts on blocking CCR5 function have focused on deleting the CCR5 gene *ex vivo* by several gene editing technologies, including CRISPR and zinc finger nuclease (ZFN) proteins. Genome editing of the HIV co-receptor CCR5 by CRISPR-Cas9 protects CD4^+^ T cells from HIV-1 infection [58]. A completed Phase I clinical trial study (2015) was carried out to determine whether “zinc finger” modified CD4^+^ T-cells are safe to give to humans and how the procedure would affect HIV-1 status (www.clinicaltrials.gov). Another clinical trial on CCR5-modified CD4^+^ T cells for HIV infection is about to start in mid-December 2018.

### CCR5 Inhibitors and cART

The success of current cART therapies is limited by the emergence of drug-resistance, potential drug toxicity, the need for sustained adherence and costs. Advances in cART have generally resulted in reduced viral spread, but not in full viral clearance. There are numerous ongoing efforts to explore the most effective ways to intensify standard cART activity [59–61] and to more greatly impact ongoing viral propagation. Most recent efforts include combined therapies targeting reservoir reduction by a combination of cART and CCR5 blockers, due to an establishment of fewer or smaller reservoirs and a concomitant reduction in residual viral replication [62,63]. In addition, association of heterozygous CCR5Δ32 deletion with survival in HIV-infection revealed the protective role of CCR5Δ32 and extends it to the long-term survival in a large cohort of HIV-1 infected patients. Not only that CCR5Δ32 demonstrates its noticable antiretroviral effect, but it also enhances the long-term survival of patients on cART [64].

## Conclusion

CCR5 blockers have great therapeutic potential for prevention and treatment of HIV-1 infection and perhaps (and importantly) a reduction of establishment, size, and/or persistence of reservoirs of latent HIV-1. Due to the potential beneficial effects of CCR5 inhibitors, their inclusion in clinical regimens may offer new possibilities for treating HIV-1 infection and associated disease.
